# Effect of orthopaedic resident education on screening for intimate partner violence

**DOI:** 10.1186/s40621-021-00355-5

**Published:** 2021-10-29

**Authors:** Mikaela J. Peters, Darren M. Roffey, Kelly A. Lefaivre

**Affiliations:** 1 Department of Orthopaedics, Faculty of Medicine, University of British Columbia: Diamond Health Care Centre, 11th Floor - 2775 Laurel Street, Vancouver, BC V5Z 1M9 Canada; 2 Division of Orthopaedic Trauma, Vancouver General Hospital, Vancouver Coastal Health: Diamond Health Care Centre, 3rd Floor - 2775 Laurel Street, Vancouver, BC V5Z 1M9 Canada

**Keywords:** Intimate partner violence, Residency, Orthopaedic, Education, Mentorship, Classroom, Fracture

## Abstract

**Background:**

Intimate Partner Violence (IPV) is prevalent in women presenting to orthopaedic fracture clinics. Rates of IPV have increased during the COVID-19 global pandemic. Our aim was to determine the effect of educational experiences on IPV knowledge and IPV screening to inform best-practices in resident education.

**Methods:**

Cross-sectional online survey of orthopaedic surgery residency programs in Canada. Demographics, IPV educational experiences, IPV knowledge, and frequency of IPV screening were collected via a modified version of the Physician Readiness to Manage Intimate Partner Violence Survey (PREMIS). Descriptive statistics and regression modeling identified predictors of IPV knowledge and frequency of IPV screening.

**Results:**

Responses were obtained from 105 orthopaedic residents; 84% participated in classroom training, 39% underwent mentorship training, 32% received both classroom training and mentorship, and 10% reported neither. Classroom training had no statistically significant association with IPV knowledge or frequency of IPV screening. Residents who received mentorship were 4.1 times more likely to screen for IPV (95% CI: 1.72–10.05), older residents were more likely to screen for IPV (OR: 8.3, 95% CI: 2.64–29.84), and senior residents were less likely to screen for IPV than junior residents (OR: 0.29, 95% CI: 0.09–0.82).

**Conclusions:**

Classroom training was not associated with any effect on IPV knowledge nor the frequency of IPV screening. Educational efforts should be targeted at increasing mentorship opportunities in order to improve IPV screening practices in Canadian orthopaedic residents.

## Background

The World Health Organization defines intimate partner violence (IPV) as physical, sexual, and emotional abuse and controlling behaviours by an intimate partner (World Health Organization 2012). IPV is a significant determinant of morbidity and mortality. After head and neck injuries, musculoskeletal injuries are the second most prevalent physical manifestation of IPV (Bhandari et al. 2006).

Findings from a cross-sectional, multi-center study of orthopaedic fracture clinics in Canada, the USA, the Netherlands, Denmark, and India indicated that 1 in 6 women disclosed a history of IPV within the last year, and 1 in 3 had experienced IPV in their lifetime (PRAISE Investigators et al. 2013). In 2020, the United Nations issued a warning that IPV rates were rising because of the global pandemic caused by the Coronavirus disease (COVID-19)—due in large part to staying at home in compliance with quarantine requirements. Increasing volumes of calls to support crisis lines have also been reported worldwide, in conjunction with decreasing urgent care seeking (Kumar 2020; Bradley et al. 2020; Muldoon et al. 2021).

Medical societies including the American College of Surgeons (2018), the World Health Organization (2013) and the Canadian Orthopaedic Association (2019) have published position statements recognizing the importance of identifying IPV. However, studies have shown that orthopaedic surgery staff and residents have misperceptions about IPV, and many do not feel they have the knowledge or resources to effectively screen their patients for IPV (Sprague et al. 2013; Della Rocca et al. 2013).

Education programs targeted towards orthopaedic fracture clinics exist and have been shown to improve IPV knowledge in the short term (EDUCATE Investigators 2018; Madden et al. 2015). Alas, the evidence is mixed as to the effect on screening; a 1-h educational program administered to trauma residents did not change the number of patients screened for IPV, nor did it change the number of IPV cases identified over a 6-month period (Sims et al. 2011). A novel IPV education program consisting of a video, didactic lecture, and case-based learning improved knowledge, comfort and preparedness in internal medicine residents, but screening practices were not evaluated (Insetta and Christmas 2020). Mentorship has been shown to correlate with IPV screening in the nursing literature (Githui et al. 2018; Taft et al. 2015), but to our knowledge, this has not been specifically examined with respect to physicians. Literature from other medical specialties suggests that multi-modal training with system-wide screening measures are required to produce long-term change in IPV screening practices (Ambuel et al. 2013; Lapidus et al. 2002).

Anecdotal evidence from the authors’ own residency experiences and informal discussions with peers suggests that orthopaedic surgery residents in Canada may not receive adequate training regarding screening for IPV. The aim of our study was to determine the effect of educational experiences on IPV knowledge and IPV screening practices of Canadian orthopaedic surgery residents. We believe this information is important to gather to help guide the creation of IPV training resources in orthopaedic surgery residency programs across Canada.

## Methods

### Study design

This was a cross-sectional study conducted in coordination with the 17 orthopaedic residency training programs across Canada. All Canadian orthopaedic surgery residents were invited to participate voluntarily in an online survey via email after receiving an invitation from their local program administrator. Study data were collected anonymously from participants between January 2, 2020 to February 3, 2020 using the University of British Columbia (UBC) Survey Tool provided by Qualtrics (Qualtrics, Provo, UT) (Bosch 2005). The Survey Tool is a cloud-based service provisioned by Qualtrics, a service provider contracted by UBC. The Survey Tool meets all security requirements set forth by the UBC Behavioural Research Ethics Board. The study protocol was approved by the UBC Behavioural Research Ethics Board (H19-03510) and the research was conducted in accordance with the Strengthening the Reporting of Observational Studies in Epidemiology (STROBE) guidelines. Informed consent was obtained in the context of administering the survey.

### Outcome measurements

The Physician Readiness to Manage IPV Survey (i.e. PREMIS) was used to evaluate comfort with IPV screening, IPV screening practices, and IPV knowledge (Short et al. 2006). This 15-min self-administered questionnaire consists of four sub-scores regarding IPV: perceived knowledge, actual knowledge, perceptions about readiness to manage, and frequency of screening. Responses were collected using a combination of a 5-point Likert scale, closed (yes/no) questions, and open-ended questions. The PREMIS has been shown to have good internal consistency, reliability and correlation with measured office IPV practices (Short et al. 2006). The actual knowledge subscale has been used in the past to evaluate the effect of formal IPV training in orthopaedic surgeons (Della Rocca et al. 2013). It was determined a priori that our primary outcome variables would be: (1) frequency of IPV screening and; (2) IPV actual knowledge.

In order to evaluate mentorship in the residency setting, we added an additional question to the PREMIS survey, namely: “What IPV screening have you experienced in residency?”. Response options included “Witnessed a preceptor screening”, “Direct teaching about screening from a preceptor”, “Fracture clinic with an established screening protocol”, and “Have not witnessed any screening”, as well as a free-text response box. Demographic information was also collected, including: age, gender, year of residency (postgraduate-years (PGY) 1–5), and overall size of the orthopaedic surgery residency program. Additionally, participants estimated the total number of hours of prior IPV teaching they had received.

Classroom training was subcategorized into self education, structured education, or didactic education. Self-education included reading institutional IPV protocols or watching videos about IPV screening. Structured education included participating in skills-based training, in-depth training > 4 h, or continuing medical education (CME) training. Didactic education included participating in a lecture or talk, or medical school classroom training. Mentorship was sub-categorized as observational or immersive mentorship. Observational mentorship was defined as working in a fracture clinic with an established screening protocol or witnessing a preceptor screening. Immersive mentorship was defined as receiving direct teaching from a preceptor about IPV screening. For the purposes of analysis, educational experiences—as identified by the survey—were grouped into classroom training or mentorship.

### Data preparation and statistical analysis

The PREMIS raw data was scored in an Excel spreadsheet (Microsoft, USA) according to the algorithm published by the developer (Short et al. 2006). Actual IPV knowledge and hours of previous training were treated as continuous variables. Age, gender, frequency of IPV screening, mentorship, classroom training, level of training, and program size were treated as categorical variables. Statistical analysis was completed using the R programming language (R Foundation for Statistical Computing, USA).

#### IPV actual knowledge: Single variable descriptive summaries, plots and linear regression.

Each categorical predictor variable was explored separately using side-by-side boxplots and summary statistics by level. A linear regression model was fit and analysis of variance (ANOVA) of the model was done to test the effect of the predictor on IPV actual knowledge score. The non-parametric Kruskal–Wallis rank sum test was also performed. Hours of previous training was explored with a scatterplot and Spearman’s rank correlation (rho). A linear regression model was fit, and ANOVA of the model done to test for a relationship of this predictor with IPV actual knowledge score.

#### IPV actual knowledge: Multiple variable descriptive summaries, plots and linear regression.

Interactions were explored between each predictor variable with classroom training and also with mentorship separately. The interaction between classroom training and mentorship was also explored. An ANOVA for each of the linear regression interaction models tested the interaction effect on IPV actual knowledge score.

#### Frequency of IPV screening: Single variable descriptive summaries, plots and ordinal logistic regression

Single variable plots and summaries were provided such that frequency of screening was analyzed as an ordinal outcome variable with three levels. An asymptotic linear-by-linear association test was done for each non-ordered nominal predictor variable with frequency of screening, the ordered nominal variable. An ordinal logistic regression model was fit and analysis of deviance of the model done to test the effect of the predictor.

#### Frequency of IPV screening: Multiple variable ordinal logistic regression, main effects only

Ordinal logistic regression models were fit to assess the effects of classroom training and mentorship separately where adjustments for all other predictor variables were included initially. Backward elimination was performed where the most insignificant predictors were removed iteratively. This was repeated excluding hours of previous training in the initial full model and resulted in the same reduced model. Further multiple variable models are considered that included classroom training and mentorship together.

## Results

A total of 105 survey responses were obtained out of a total of 331 orthopaedic residents in Canada in 2020, representing a participation rate of 31.7%. There were 57 residents who identified as male (54%), while 68 were classified as junior residents (65%). Most residents (90%) were aged between 31 and 40 years old. Participant demographics are presented in Table 1.

**Table 1:** Demographic Information

Demographic	Category	*n*	%
Age (Years)	26–30	5	5
	31–35	72	69
	36–40	22	21
	41–45	6	6
Gender	Male	57	54
	Female	48	46
Year of residency	PGY1	24	23
	PGY2	20	19
	PGY3	24	23
	PGY4	20	19
	PGY5	17	16
Junior resident	PGY1-3	68	65
Senior resident	PGY4-5	37	35
Number of residents in the program	0–10	8	8
	11–20	38	36
	21–30	41	39
	31–40	5	5
	41 +	13	12

PGY: Postgraduate-year

The mean IPV actual knowledge score was 27.9 out of a maximum of 38. Residents reported a mean of 3.7 h of IPV screening training. The mean response to frequency of IPV screening was 2.4—equating to approximately mid-way between “Seldom” and “Sometimes”. PREMIS subscale scores and resident training logs are further described in Table 2.

**Table 2:** PREMIS subscale scores and resident training logs

PREMIS Subscale Scores	Mean	SD	Range (Min–Max)
Perceived Preparation *Lowest possible score: 1; Highest possible score: 7*	3.2	1.1	1.0–6.0
Perceived Knowledge *Lowest possible score: 1; Highest possible score: 7*	3.3	1.0	1.4–5.9
Actual Knowledge Score *Lowest possible score: 0; Highest possible score: 38*	27.9	5.0	2.0–34.0
Resident Training Logs			
Hours of IPV screening training received *Lowest possible score: 0; Highest possible score: 20*	3.7	4.4	0.0–20.0
Frequency of screening (1–5) *Lowest possible score: 1; Highest possible score: 5*	2.4	1.5	1.0–4.0

*PREMIS* Physician Readiness to Manage Intimate Partner Violence Survey, *IPV* Intimate Partner Violence, *SD* Standard Deviation

Residents received varying education experiences pertaining to IPV: 88 (84%) reported participating in classroom training, whereas only 40 (39%) had received mentorship; 34 (32%) residents reported both forms of educational experiences. Meanwhile, 11 participants (10%) reported that they had received no prior classroom training or mentoring about IPV screening. Resident training is further described in Table 3.

**Table 3:** Resident Education Experiences

	n	%
Classroom Training	88	84
Self Education	21	20
*Read institution's IPV protocol or watched a video about IPV screening*		
Structured Education	10	10
*Participated in skills-based training, in-depth training* > *4 h, or CME training*		
Didactic Education	81	77
*Participated in a lecture or talk, or medical school classroom training*		
Mentorship	40	39
Observational Mentorship	28	27
*Worked in a fracture clinic with an established screening protocol, or*		
*Witnessed a preceptor screening*		
Immersive Mentorship	27	26
*Received direct teaching from a preceptor about training*		
Classroom Training AND Mentorship	34	32
No Training	11	10

*CME* continuing medical education, *IPV* intimate partner violence

### IPV actual knowledge

Multivariable linear models fit to assess the effects of classroom training and mentorship on IPV actual knowledge indicated there were no significant effects. The effect of classroom training and mentorship alone cannot be determined since there are cases that have both for this outcome. Therefore, further multiple variable models were considered that included these together as well. Backward elimination was performed on these models and no significant effects on IPV actual knowledge score were indicated.

### Frequency of IPV screening

Asymptotic linear-by-linear association tests were statistically significant for frequency of IPV screening (ordered) by age (26–35 vs. 36–45 years of age) (statistic: 8.898; *p* = 0.003) and by mentorship (no vs. yes) (statistic: 10.33; *p* = 0.001). Multiple variable ordinal logistic regression models showed that classroom training had no statistically significant impact on frequency of screening (OR 2.5, 95% CI 0.83–8.14). Residents who received mentorship were 4.1 times more likely to screen for IPV (95% CI: 1.72–10.05). Older residents (> 35 years old) were more likely to screen for IPV than younger residents (OR: 8.3, 95% CI: 2.64–29.84). Senior residents were less likely to screen for IPV than junior residents (OR: 0.29, CI: 0.09–0.82). Odds ratios for the model fit to frequency of IPV screening are summarized in Table 4.

**Table 4:** Odds ratios for frequency of IPV screening

	OR	95% CI
Classroom training	2.51	0.83–8.13
Mentorship	4.08	1.72–10.05
Senior Resident	0.29	0.09–0.82
Age > 35 Years	8.31	2.64–29.84

*OR* Odds ratio, *CI* Confidence interval

## Discussion

IPV is an escalating concern for the health and wellness of women worldwide; 1 in 6 women presenting to orthopaedic trauma clinics has experienced IPV within the last year, and 1 in 3 in their lifetime (PRAISE Investigators et al. 2013). The incidences of IPV are increasing as a result of the COVID-19 pandemic (Lyons and Brewer 2021), with many regions reporting significant increases in the number of calls to support services (Sánchez et al. 2020). Equally concerning are the regions where calls to IPV hotlines have decreased dramatically, which IPV experts believe is due to the fact that women are unable to safely connect with support services on account of their partner denying them access (Evans et al. 2020). As a consequence, screening in healthcare settings, in particular those involving interactions with orthopaedic clinicians, may be one of the only feasible opportunities to identify such women.

In recent years, medical societies have published position statements highlighting the importance of IPV (World Health Organization 2012; American College of Surgeons 2018; Canadian Orthopaedic Association 2019). Alas, Sprague et al. (2013) found that only 39.7% of surgical residents reported any IPV education. The results from our survey demonstrated that a majority of orthopaedic surgery residents in Canada are now receiving IPV education, with 90% of respondents indicating that they have received some form of IPV education—therein suggesting that significant improvements have been made in medical education. Unfortunately, our findings suggest that training alone is not sufficient to promote IPV screening.

We chose to focus on self-reported screening frequency as the primary outcome as this has the potential to have the highest impact on patient care. We know that the prevalence of IPV is high when presenting in fracture clinics, but a relatively small proportion of individuals present with injuries directly related to IPV (Bhandari et al. 2006; Bradley et al. 2020). This notion is supported by the fact that IPV victims commonly present with non-violent mechanisms of injury (Madden et al. 2015). Screening for IPV based on reported mechanism of injury is not effective; universal screening is required.

It has been previously demonstrated that IPV training typically improves scores on a written knowledge test about IPV. Madden et al. (2015) found that a half day course on IPV improved orthopaedic surgery trainee’s knowledge at 3 months post-education. This was repeated with a larger cohort by the EDUCATE Investigators (2018), who also demonstrated increased knowledge scores at 3 months post-education. In contrast to the prior literature, our results indicated no statistically significant predictors of the IPV actual knowledge score; the effects of mentorship or classroom training were not significant. Further, there were no significant differences in IPV actual knowledge scores with respect to gender, level of training, age, program size, or hours of previous training. Since the proportion of our cohort with some form of IPV education was quite high, and our sample size was relatively small, our data set may not have had adequate power to identify significant differences in knowledge scores.

A lack of association between classroom training and IPV screening practices has been previously observed in the trauma literature by Sims et al. (2011). In their study, a group of trauma residents received IPV screening education in the form of a 1-h conference, and a chart review was used to compare documentation of IPV screening pre- and post-education. Although there was a mild increase in knowledge scores on a written test, there was no difference in documentation in charts.

The field of implementation research examines the translation of knowledge into practice. In their summary of this body of literature, Fixsen et al., (2005) emphasize the importance of implementation in the context of community. Strong leadership, shared goals, and effective coaching all improve adherence to new evidence-based practices in healthcare settings (Fixsen et al. 2005). Other models for quality improvement projects in healthcare settings similarly include both traditional training and an element of coaching or mentorship for physicians (Li et al. 2015; Williams et al. 2014). In the emergency and primary care settings, multi-modal education programs have been used to improve IPV screening practices. Ambuel et al., (2013) used two levels of education: a small group of individuals in management positions received 20 h of training that included strategies for developing procedural change in clinics, while individuals in direct contact with patients received a 3-h training program. While knowledge scores were no different 2 years post-intervention, the program was successful in increasing the number of patients with IPV screening documented in the chart.

It has been documented that lack of time, lack of knowledge of what to ask, and lack of knowledge of community resources are significant barriers to screening in medical students and surgical residents (Sprague et al. 2013). We hypothesize that mentorship can be helpful in demonstrating that screening for IPV can be done easily without a significant time investment. Clear procedures in place for next steps when a positive case is identified, such as in a fracture clinic with an established screening protocol, can further reduce barriers.

Curiously, we found that junior residents were more likely to screen for IPV than senior residents. We hypothesize that this may be related to the type of work done by each resident, with junior residents being more likely to see new patients in the emergency department. Interestingly, residents over the age of 35 years were more likely to screen than younger residents. Further research is required to delineate the types of situations in which screening occurs to further explore why older residents are more inclined to screen for IPV.

Mentorship was highly effective at improving IPV screening practices in our study, and the majority of residents reported some form of classroom training. Unfortunately, only 28 residents (27%) reported working in a fracture clinic with an established screening protocol or witnessing a preceptor screening. Together, these results highlight the need for a system-wide change within Canadian orthopaedic resident training in order to effectively increase IPV screening.

## Limitations

The survey response rate from residents was low (31.7%), leading to the potential for non-response bias. There may also be an element of selection bias, as residents with an interest in IPV could have self-selected and completed the survey. The fact that 46% of responders identified as female, while only 27% of orthopaedic residents in Canada in 2019–20 were females, indicates that the results may be biased due to more females responding (Canadian Post-M.D. Education Registry 2020). As this was a cross-sectional study, there could be an issue with recall bias across respondents.

We used a 5-point Likert scale for determining frequency of IPV screening, but only the lower 4 points of the scale were used, potentially limiting the sensitivity of this tool. We did not collect data on when previous IPV education had occurred during the residency period. Also, further study is required about the attrition of IPV knowledge over time. The potential for variability in both classroom training and mentorship at universities across Canada was difficult to take into consideration.

We acknowledge that our study design (i.e. online survey) provides primary findings. To determine the effect of various types of education on IPV knowledge and IPV screening, the ‘gold standard’ would be to conduct a randomized controlled trial, in which participants are randomized to mentorship, classroom training, or no education, with the levels of change in IPV knowledge and IPV screening compared across the three treatment groups over time. Obviously, this type of trial is not feasible in the context of residency education. Thus, our results can be used to illuminate the current climate amidst increased awareness and scrutiny with respect to the impact of the COVID-19 global pandemic on IPV.

## Conclusions

Mentorship was associated with an increased likelihood of screening for IPV and should be a focus of any remediation of the educational experiences incorporated into residency programs. Classroom training was not associated with IPV actual knowledge or frequency of IPV screening. Further study is required to identify the optimal, multi-modal, interdisciplinary training with which to improve IPV knowledge and IPV screening practices by Canadian orthopaedic residents.

## Data Availability

All data generated or analysed during this study are included in this published article.

## Abbreviations

IPV: Intimate Partner Violence
PREMIS: Physician Readiness to Manage Intimate Partner Violence Survey
CI: Confidence Interval
OR: Odds Ratio
UBC: University of British Columbia
STROBE: Strengthening the Reporting of Observational Studies in Epidemiology
PGY: Postgraduate-year
CME: Continuing Medical Education
ANOVA: Analysis of Variance
n: Number
%: Percentage
SD: Standard Deviation
P: Probability
